# Supplementary material for: An open-source framework for large-scale, flexible evaluation of biomedical text mining systems
Source: J Biomed Discov Collab. 2008 Jan 29;3:1. doi: 10.1186/1747-5333-3-1 (PMC2276192; doi:10.1186/1747-5333-3-1)
Supplement: Additional file 1 — Evaluation platform source code. The additional file contains the software evaluation framework discussed in this paper. The most current release can be downloaded from SourceForge.net [1]. See the accompanying README file for instructions on installation and use. Also included in the distribution is a collection of UIMA wrappers for some commonly used BioNLP tools and annotated corpora. [file 1747-5333-3-1-S1.zip › bionlp_uima_repository-1.0.1/docs/api/src-html/edu/uchsc/ccp/uima/mention/CCPMention_Type.html]

```
001    
002    /* First created by JCasGen Tue Aug 28 16:44:10 MDT 2007 */
003    package edu.uchsc.ccp.uima.mention;
004    
005    import org.apache.uima.cas.Feature;
006    import org.apache.uima.cas.FeatureStructure;
007    import org.apache.uima.cas.Type;
008    import org.apache.uima.cas.impl.CASImpl;
009    import org.apache.uima.cas.impl.FSGenerator;
010    import org.apache.uima.cas.impl.FeatureImpl;
011    import org.apache.uima.cas.impl.TypeImpl;
012    import org.apache.uima.jcas.JCas;
013    import org.apache.uima.jcas.JCasRegistry;
014    import org.apache.uima.jcas.cas.TOP_Type;
015    
016    /** The superclass for all CCP Mentions (class mention, complex slot mention, and non-complex slot mention)
017     * Updated by JCasGen Tue Aug 28 16:44:10 MDT 2007
018     * @generated */
019    public class CCPMention_Type extends TOP_Type {
020      /** @generated */
021      protected FSGenerator getFSGenerator() {return fsGenerator;}
022      /** @generated */
023      private final FSGenerator fsGenerator = 
024        new FSGenerator() {
025          public FeatureStructure createFS(int addr, CASImpl cas) {
026                             if (CCPMention_Type.this.useExistingInstance) {
027                               // Return eq fs instance if already created
028                         FeatureStructure fs = CCPMention_Type.this.jcas.getJfsFromCaddr(addr);
029                         if (null == fs) {
030                           fs = new CCPMention(addr, CCPMention_Type.this);
031                               CCPMention_Type.this.jcas.putJfsFromCaddr(addr, fs);
032                               return fs;
033                         }
034                         return fs;
035            } else return new CCPMention(addr, CCPMention_Type.this);
036              }
037        };
038      /** @generated */
039      public final static int typeIndexID = CCPMention.typeIndexID;
040      /** @generated 
041         @modifiable */
042      public final static boolean featOkTst = JCasRegistry.getFeatOkTst("edu.uchsc.ccp.uima.mention.CCPMention");
043     
044      /** @generated */
045      final Feature casFeat_mentionName;
046      /** @generated */
047      final int     casFeatCode_mentionName;
048      /** @generated */ 
049      public String getMentionName(int addr) {
050            if (featOkTst && casFeat_mentionName == null)
051          jcas.throwFeatMissing("mentionName", "edu.uchsc.ccp.uima.mention.CCPMention");
052        return ll_cas.ll_getStringValue(addr, casFeatCode_mentionName);
053      }
054      /** @generated */    
055      public void setMentionName(int addr, String v) {
056            if (featOkTst && casFeat_mentionName == null)
057          jcas.throwFeatMissing("mentionName", "edu.uchsc.ccp.uima.mention.CCPMention");
058        ll_cas.ll_setStringValue(addr, casFeatCode_mentionName, v);}
059        
060      
061    
062    
063    
064      /** initialize variables to correspond with Cas Type and Features
065            * @generated */
066      public CCPMention_Type(JCas jcas, Type casType) {
067        super(jcas, casType);
068        casImpl.getFSClassRegistry().addGeneratorForType((TypeImpl)this.casType, getFSGenerator());
069    
070     
071        casFeat_mentionName = jcas.getRequiredFeatureDE(casType, "mentionName", "uima.cas.String", featOkTst);
072        casFeatCode_mentionName  = (null == casFeat_mentionName) ? JCas.INVALID_FEATURE_CODE : ((FeatureImpl)casFeat_mentionName).getCode();
073    
074      }
075    }
076    
077    
078    
079
```
